# The association between neighborhood greenness and cardiovascular disease: an observational study

**DOI:** 10.1186/1471-2458-12-466

**Published:** 2012-06-21

**Authors:** Gavin Pereira, Sarah Foster, Karen Martin, Hayley Christian, Bryan J Boruff, Matthew Knuiman, Billie Giles-Corti

**Affiliations:** 1 Telethon Institute for Child Health Research, Centre for Child Health Research, The University of Western Australia, 100 Roberts Road, Subiaco, WA, 6008, Australia; 2 Centre for the Built Environment and Health, School of Population Health, The University of Western Australia, 35 Stirling Highway, Crawley, WA, 6009, Australia; 3 School of Earth and Environment, The University of Western Australia, 35 Stirling Highway, Crawley, WA, 6009, Australia; 4 School of Population Health, The University of Western Australia, 35 Stirling Highway, Crawley, WA 6009, Australia; 5 McCaughey VicHealth Centre for the Promotion of Mental Health and Community Wellbeing, Melbourne School of Population Health, The University of Melbourne, 207 Bouverie Street, Melbourne, Victoria 3010, Australia

**Keywords:** Coronary heart disease, Stroke, Cardiovascular disease, Greenness, Built environment

## Abstract

**Background:**

Previous studies have demonstrated links between cardiovascular disease and physical inactivity and poor air quality, which are both associated with neighborhood greenness. However, no studies have directly investigated neighborhood greenness in relation to coronary heart disease risk. We investigated the effect of neighborhood greenness on both self-reported and hospital admissions of coronary heart disease or stroke, accounting for ambient air quality, socio-demographic, behavioral and biological factors.

**Method:**

Cross-sectional study of 11,404 adults obtained from a population representative sample for the period 2003–2009 in Perth, Western Australia. Neighborhood greenness was ascertained for a 1600 m service area surrounding the residential address using the mean and standard deviation of the Normalized Difference Vegetation Index (NDVI) obtained from remote sensing. Logistic regression was used to assess associations with medically diagnosed and hospitalization for coronary heart disease or stroke.

**Results:**

The odds of hospitalization for heart disease or stroke was 37% (95% CI: 8%, 57%) lower among adults in neighborhoods with highly variable greenness (highest tertile) compared to those in predominantly green, or predominantly non-green neighborhoods (lowest tertile). This effect was independent of the absolute levels of neighborhood greenness. There was weaker evidence for associations with the mean level of neighborhood greenness.

**Conclusion:**

Variability in neighborhood greenness is a single metric that encapsulates two potential promoters of physical activity - an aesthetically pleasing natural environment and access to urban destinations. Variability in greenness within a neighborhood was negatively associated with coronary heart disease and stroke.

## Background

More people die annually from heart disease than any other cause and the World Health Organization projects that cardiovascular disease will remain the leading single cause of death by 2030 [1]. The majority of cardiovascular mortality can be attributed to coronary heart disease and stroke which together account for 18–20% of the total disease burden in Australia [2] and United States [3]. Although age, sex, family history and ethnicity are strong risk factors for heart disease, it is the modifiable risk factors that have the greatest potential for improved public health [4]. Hypertension, abnormal blood lipids, diabetes, unhealthy diet, physical inactivity, smoking and inappropriate alcohol consumption have been identified as the most prevalent modifiable risk factors[4].

The link between physical activity and the physical environment is becoming more well-established [5-7]. Increased physical activity has been associated with both proximity to parks [8,9] and greater land-use mix [10]. However, the role that the built and natural environment plays in explaining cardiovascular morbidity is less well understood. Various studies have examined the association between green neighborhoods and body weight, which is an intermediate outcome on the pathway to heart disease. However, few studies have explicitly assessed its association with cardiovascular outcomes, despite the potential for greener neighborhoods to promote physical activity. We hypothesized that mean neighborhood greenness (measured by remote sensing) was negatively associated with both hospital admission and self-reported medically diagnosed coronary heart disease or stroke.

## Methods

### Study design and participants

A cross-sectional study was undertaken for 11,404 adults aged 25 and over who (i) were residents of the Perth metropolitan area, (ii) consented to data linkage and (iii) completed the Western Australian Health and Wellbeing Survey between 2003 and 2009. This monthly computer-assisted telephone interview was administered by the Western Australian Department of Health and responses were obtained for a stratified random sample of the state population (N = 1,959,088; 2006 Census). There were 15,502 adult residents in Perth who completed the survey between 2003 and 2009. Of those participants, 11,404 (74%) participants granted permission for data linkage.

### Outcome variables

Self-report of prior medically diagnosed heart disease and stroke was obtained from the Health and Wellbeing Survey. Hospital records were obtained from the Western Australian Department of Health. Coronary heart disease and stroke were identified from these records as a primary diagnosis coded I20 – I25 and I60 – I68 according to the International Classification of Diseases (ICD 10). Participants were considered to have been hospitalized for heart disease if the admission occurred within a three year window centered on the year that the participant completed the Health and Wellbeing Survey.

### Greenness

Greenness was measured using the Normalized Difference Vegetation Index (NDVI) derived from annually updated Landsat TM remote sensing imagery taken during summer. NDVI provides an indication of the presence and condition of green vegetation with values typically ranging from −1 to +1. Values of −1 generally represent water, while values of zero (−0.1 to 0.1) correspond to bare surfaces such as rock, sand, rooftops and roads. Higher values (0.2 to 0.4) represent grassland or bush land and values of +1 represent healthy green vegetation [11]. Water features were first removed before the NDVI was calculated.

Neighborhoods around participants’ homes were defined using 1600 m (network distance) service areas. The rationale for selecting a 1600 m service area was based on the assumption that physical activity is the most likely pathway by which neighborhood greenness is associated with cardiovascular disease risk. A 1600 m service area represents how far a participant could walk from home at a moderate to vigorous intensity pace, within 15 minutes, which equates to half the recommended level of daily physical activity for adults[12]. That is, the daily recommended level of physical activity of 30 minutes would be attained for a return trip. Moreover, a 1600 m service area has been shown to be a critical distance for examining the relationship between parks and walking. Sugiyama *et al.* found that although proximity to parks was generally associated with walking, it was the presence of parks within 1600 m that was most associated with sufficient walking (>150 minutes/week)[9].

**Figure 1:**
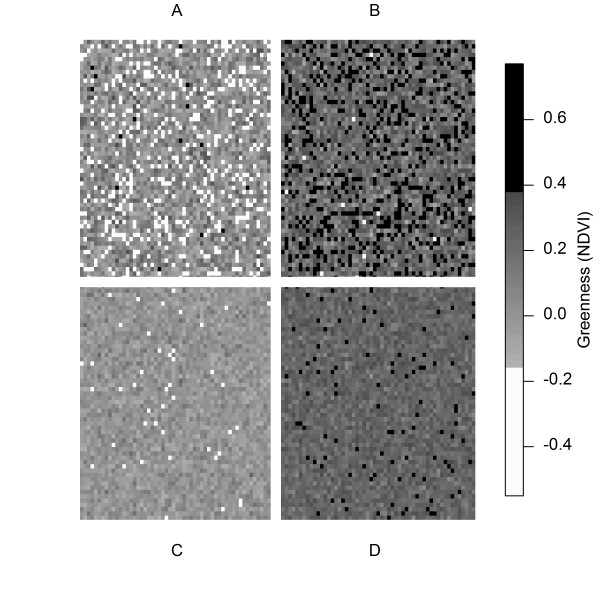
**Four simulated service areas.** Area A has low mean but high variability (standard deviation) in greenness. Area B has both high mean and variability in greenness. Area C has both low mean and variability in greenness. Area D has high mean but low variability in greenness. In general, the prevalence of high NDVI pixels illustrates mean greenness, and the prevalence of contrasting high and low NDVI pixels illustrates variability in greenness.

**Figure 2:**
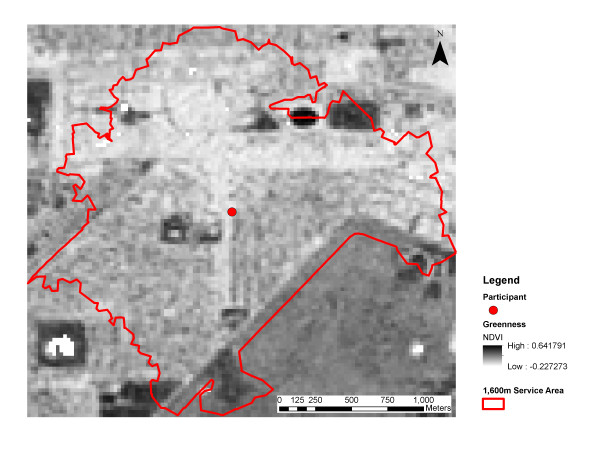
**Illustration of a service area with a high variability in greenness (NDVI Mean 0.04, SD 0.13).** The lighter areas represent lakes, roads and buildings. Darker areas represent parks, ovals and bush land. The white section with east–west alignment at the north side of the service area and the white section with north–south alignment in the middle of the service area is commercial land. The remaining area is predominantly residential. A large section of bush land lies outside and south-east of the service area.

**Figure 3:**
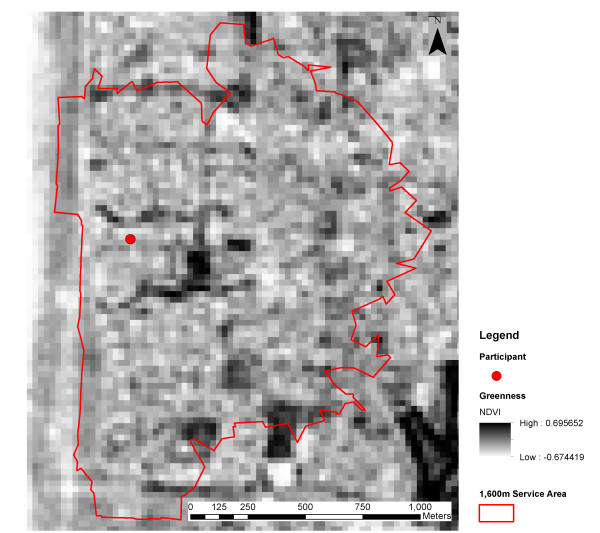
**Illustration of a service area with low variability in greenness (NDVI Mean 0.10, SD 0.08).** The lighter areas represent lakes, roads and buildings. Darker areas represent parks, ovals and bush land. The figure is more uniform in terms of NDVI. The Indian Ocean lies outside and to the west of the service area. This service area has a lower spatial variability (i.e. predominantly high levels and few low levels) of greenness than Figure 1.

The mean and standard deviation of NDVI values were calculated for the 1600 m service areas. The simulation shown in Figure 1 demonstrates that the mean NDVI describes the absolute level of greenness, while the standard deviation of NDVI captures the heterogeneity in the distribution of greenness. In reality, the green and non-green areas (pixels) are not randomly distributed as they cluster, such as in parks or along roads. Figures 2 and 3 illustrate the spatial variability in greenness for two study participants and their respective service areas. The standard deviation of NDVI values within the service area can be interpreted as an alternative expression of land-use mix of anthropogenic and natural features. The service area in Figure 2 indicates lower levels of greenness than the service area in Figure 3 but has a higher level of variability. In general, high levels of variability in greenness will occur when the neighborhood contains both high NDVI (green) areas and low NDVI (non-green areas). In this example, the higher level of variability is due to both the strong prevalence of vegetation (high NDVI) and the presence of commercial land-use (low NDVI).

The mean and standard deviation of NDVI values were analyzed as both continuous linear variables (scaled by their interquartile range) and categorical variables (tertiles). The rationale for using tertiles is provided in the *Supplementary Material* (Additional file 1). Briefly, cut-points at tertiles provided a compromise between capturing the pattern of the association and ensuring sufficient data within each category. Tertiles also provide an equal amount of data in each category and allow interpretation relative to “low”, “medium” and “high” values.

### Statistical analysis

Adjustments were made using representations of a range of well-established factors [4] obtained from the Health and Wellbeing Survey: sociodemographics (age, sex, possession of a healthcare card, education, household income), biological factors (non-gestational diabetes, BMI, hypertension, high cholesterol), behavioral factors (daily serves of fruit and vegetables, risky drinking in the last month (>6 standard drinks for men, >4 standard drinks for women), and smoking (never versus ever smoked)), and a proxy for air quality.

A broad class of anthropogenic air pollutants have been causally linked with endothelial dysfunction and vasoconstriction, increased blood pressure, systemic inflammatory responses, oxidative stress, and the progression of atherosclerosis [13]. Traffic-related air pollution specifically has been recently associated with fatal and non-fatal coronary heart disease events [14]. In a synthesis of studies conducted by the Health Effects Institute it was concluded that the exposure zone for traffic-related air pollution is between 300 m and 500 m from a major road, beyond which concentrations decrease to background levels [15]. Adjustment was made for the total length of main roads within a 400 m service area as a proxy for exposure to ambient air pollution. Main roads were defined as all roads traversed by ≥6000 vehicles/day. The metropolitan road network was obtained from Landgate (the state government source of land information and geographic data) at four time points (2005, 2006, 2008, 2009). The year of road network data was matched to participant’s year of interview for the Health and Wellbeing Survey.

Socio-demographic factors, biological factors, behavioral factors and environmental factors were cumulatively included as adjustment variables in multiple regression models. Logistic regression was applied using R 2.13 and road exposure was calculated using ArcMap 9.2.

### Ethics

Approval was obtained from the Human Research Ethics Committees of the Western Australian Department of Health and The University of Western Australia (#2010/1). This research conforms to the ethical principles for medical research of the Declaration of Helsinki.

## Results

A total of 367 (3%) adults were hospitalized for coronary heart disease or stroke and 1,415 (12%) reported a prior medical diagnosis with either condition (Table 1). The median and range for levels and variability of greenness for 1600 m service areas is shown in Table 1. The highest level of greenness (mean NDVI = 0.337) was observed for a service area that mostly contained bush land (typically eucalypt vegetation) and was located on the urban–rural fringe. The service area with the lowest level of greenness (mean NDVI = −0.059) was located in a region with a large proportion of commercial and industrial land use. The service area with the highest variability in greenness (SD NDVI = 0.205) was located in a densely populated area with large parks and bush land, a dense network of tree-lined streets and commercial and retail destinations. The lowest variability in greenness (SD NDVI = 0.048) was observed for a service area with little vegetation and large houses that aligned waterways. Complete distributions of the levels and variability in greenness are provided in the *Supplementary Material.*

**Table 1:** Distributions of risk factors for coronary heart disease or stroke among 11,404 adults resident in Perth, Western Australia who responded to the Health and Wellbeing Survey 2003-2009

	**N (%)**
*Coronary heart disease or stroke*	
Ever diagnosed	1,415 (12)
Hospitalized	367 (3)
*Sex*	
Female	6,682 (59)
Male	4,724 (41)
*Age*	
25–47 years	3,660 (32)
48–63 years	3,899 (34)
64 years and over	3,847 (34)
*Highest attained level of education*	
Less than year 10	962 (9)
Year 10 or 11	2,227 (20)
Year 12	1,212 (11)
TAFE^*a*^/Trade qualification	3,977 (36)
Tertiary degree	2,576 (24)
*Household Income*	
Less than $20,000	2,052 (20)
$20,001–40,000	2,434 (23)
$40,001–$60,000	1,667 (16)
$60,001–$80,000	1,497 (14)
More than $80,000	2,793 (27)
*Healthcare card*	
No	6,243 (59)
Yes	4,305 (41)
*Number of serves of vegetables per day*	
Less than one serve	591 (5)
One serve	1,410 (12)
Two serves	2,687 (24)
Three serves	2,678 (24)
Four serves	2,191 (19)
Five serves	1,207 (11)
Six or more serves	612 (5)
*Number of serves of fruit per day*	
Less than one serve	1,875 (16)
One serve	3,046 (27)
Two serves	3,871 (34)
Three serves	1,794 (16)
Four or more serves	809 (7)
*Number of sessions of high-risk alcohol consumption in the last 4 weeks*^*b*^	
Never	6,202 (75)
Once	527 (6)
Twice	517 (6)
Three to four times	505 (6)
Five times or more	569 (7)
*Smoking*	
Current smoker or previously smoked	4,931 (43)
Never smoked	6,474 (57)
*Total minutes of walking (transport or recreation)*	
None	3,021 (26)
1 to 90 minutes/week	3,021 (26)
91 to 200 minutes/week	2,278 (20)
More than 200 minutes/week	2,640 (23)
*Body Mass Index (BMI)*^*c*^	
Underweight (BMI < 18.5)	204 (2)
Normal weight (BMI 18.5–24.9)	4,523 (42)
Overweight (BMI 25–29.9)	4,004 (37)
Obese (BMI ≥ 30)	2,157 (20)
*Diabetes ever diagnosed*	
No	10,537 (95)
Yes	498 (5)
*High cholesterol ever diagnosed*	
No	7,273 (76)
Yes	2,291 (24)
*High blood pressure ever diagnosed*	
No	8,371 (75)
Yes	2,839 (25)
*Length of main road in 400m*^d^*service area*^*e*^*(metres)*	Median 0
	Range 0–3,898
*Level of Greenness in 1600 m service area (Mean NDVI)*	Median 0.081
	Range −0.059–0.337
*Variability of Greenness in 1600 m service area (SD NDVI)*	Median 0.103
	Range 0.048–0.205

a. TAFE: Technical and Further Education.

b. Men: >6 drinks; Women: >4 drinks. Categorized by the WA Department of Health.

c. Categorized according to WHO Technical Report Series 894 [16].

d. Distances are specified in metres(m): 400 m = 437yards, 1600 m = 1750 yards.

e. Main roads defined as >6000 vehicles/day.

### Associations with self-reported coronary heart disease or stroke

After “full adjustment”, which included terms for both mean and variability in greenness, the odds ratio for coronary heart disease or stroke (self-report) was 0.84 (95% CI: 0.69, 1.02) for adults with moderate levels of greenness (middle tertile) (Table 2). The odds ratio was 0.94 (95% CI: 0.76, 1.15) for adults with high levels of greenness (highest tertile). Stronger associations were observed for variability in greenness and effect sizes were similar for adults with moderate and high levels of variability in greenness, with a considerable overlap in the confidence intervals. The odds ratio for coronary heart disease or stroke (self-report) was 0.76 (95% CI: 0.62, 0.94) for adults in areas of moderate variability in greenness and was 0.84 (95% CI: 0.68, 1.03) for those in areas with high variability in greenness. After full adjustment, the odds ratios for a unit (interquartile range) increase in the mean levels and variability of greenness were 0.93 (95% CI: 0.85, 1.01) and 0.91 (95% CI: 0.82, 1.02) respectively. Effect estimates remained stable across adjusted models.

**Table 2:** Odds ratios (OR) and 95% confidence intervals (CI) of coronary heart disease or stroke for differences in neighborhood greenness for the 11,404 adults in the study population. Adjustment was made by cumulative inclusion of risk factors

	**Model A**	**Model B**	**Model C**	**Model D**	**Model E**	**Model F**
**Adjustment**	**No adjustment**	**Sociodemographics**	**Sociodemographics**	**Sociodemographics**	**Sociodemographics**	**Sociodemographics**
			**Biological factors**	**Biological factors**	**Biological factors**	**Biological factors**
				**Behavioral factors**	**Behavioral factors**	**Behavioral factors**
					**Air quality**	**Air quality**
						**All greenness**
**Self-reported medical diagnosis with coronary heart disease or stroke**						
Sample size (N)	11,374	9,216	7,216	5,903	5,903	5,903
*Mean greenness (NDVI) in 1600 m service area*						
Low	1	1	1	1	1	1
Moderate	0.91 (0.79, 1.05)	**0.81 (0.69, 0.96)**	**0.83 (0.69, 1.00)**	0.83 (0.68, 1.02)	0.83 (0.68, 1.02)	0.84 (0.69, 1.02)
High	1.09 (0.95, 1.24)	0.98 (0.84, 1.15)	1.01 (0.85, 1.22)	0.92 (0.75, 1.13)	0.92 (0.75, 1.13)	0.94 (0.76, 1.15)
Linear increase	0.98 (0.93, 1.04)	0.97 (0.90, 1.03)	0.98 (0.91, 1.05)	0.98 (0.93, 1.04)	0.93 (0.85, 1.01)	0.93 (0.85, 1.01)
*Standard deviation (SD) of greenness (NDVI) in 1600 m service area*						
Low	1	1	1	1	1	1
Moderate	0.84 (0.74, 0.97)	**0.71 (0.60, 0.83)**	**0.70 (0.58, 0.84)**	**0.76 (0.62, 0.93)**	**0.76 (0.62, 0.93)**	**0.76 (0.62, 0.94)**
High	0.91 (0.80, 1.04)	**0.83 (0.70, 0.97)**	**0.83 (0.69, 0.99)**	0.84 (0.68, 1.02)	0.84 (0.68, 1.03)	0.84 (0.68, 1.03)
Linear increase	0.94 (0.88, 1.01)	0.89 (0.82, 0.97)	0.90 (0.82, 0.99)	0.91 (0.82, 1.01)	0.91 (0.82, 1.01)	0.91 (0.82, 1.02)
**Hospital admission with coronary heart disease or stroke**						
Sample size (N)	11,198	8,901	6,941	5,637	5,637	5,637
*Mean greenness (NDVI) in 1600 m service area*						
Low	1	1	1	1	1	1
Moderate	1.16 (0.90, 1.50)	0.88 (0.65, 1.17)	0.87 (0.64, 1.19)	0.92 (0.65, 1.30)	0.92 (0.65, 1.30)	0.90 (0.63, 1.27)
High	1.11 (0.86, 1.44)	0.95 (0.71, 1.28)	0.82 (0.59, 1.13)	0.85 (0.58, 1.24)	0.85 (0.58, 1.24)	0.87 (0.60, 1.27)
Linear increase	0.98 (0.88, 1.08)	0.94 (0.83, 1.06)	0.89 (0.77, 1.02)	0.90 (0.77, 1.05)	0.90 (0.77, 1.05)	0.90 (0.77, 1.05)
*Standard deviation (SD) of greenness (NDVI) in 1600 m service area*						
Low	1	1	1	1	1	1
Moderate	1.01 (0.79, 1.30)	0.92 (0.69, 1.23)	0.92 (0.68, 1.24)	0.87 (0.61, 1.22)	0.85 (0.60, 1.20)	0.85 (0.60, 1.21)
High	0.90 (0.69, 1.16)	0.81 (0.60, 1.09)	**0.71 (0.51, 0.99)**	**0.66 (0.45, 0.96)**	**0.63 (0.43, 0.92)**	**0.63 (0.43, 0.92)**
Linear increase	0.94 (0.82, 1.07)	0.92 (0.79, 1.07)	0.89 (0.74, 1.05)	0.84 (0.70, 1.02)	**0.82 (0.68, 1.00)**	**0.82 (0.68, 1.00)**

Tertiles for mean greenness: Low (mean NDVI < 0.068, reference level), Moderate (0.068 ≤ mean NDVI ≤ 0.096), High (mean NDVI > 0.096).

Tertiles for standard deviation (SD) of greenness: Low (SD NDVI < 0.097, reference level), Moderate (0.097 ≤ SD NDVI ≤ 0.109), High (SD NDVI > 0.109).

Linear increase: per Inter Quartile Range (IQR) increase in mean NDVI (IQR = 0.043) and SD NDVI (IQR = 0.020).

Sociodemographics factors: age, sex, education, income, possession of a healthcare card.

Biological factors: diabetes, high cholesterol, high blood pressure, BMI.

Behavioral factors: fruit consumption, vegetable consumption, high-risk alcohol consumption, smoking.

Air quality: total length of main roads within the service area is used as a proxy for air quality.

All greenness: the model included terms for both mean and SD of NDVI.

Hospital admissions were for a 3-year period centered on the year of completion of the Health and Wellbeing Survey.

Bold text indicates statistical significance at the 5% (α) level.

### Association with hospitalization for coronary heart disease or stroke

There was weaker evidence for an association between mean greenness and hospitalization for heart disease or stroke. However after full adjustment, the odds ratio for a unit (interquartile range) increase in the mean levels was 0.90 (95% CI: 0.77, 1.05). This effect size was similar to the odds ratio for a unit increase in variability in greenness; OR 0.82 (95% CI: 0.68, 1.00). The odds ratio was 0.63 (95% CI: 0.43, 0.92) for adults with the highest variability in neighborhood greenness relative to those with the lowest variability in greenness. The effect size was intensified after additional adjustment for biological factors (Model C). That is, effect estimates were attenuated by imbalance in the data before adjustment for biological factors.

### Assessment of external generalizability

Of the 15,502 adult residents in Perth who completed the Health and Wellbeing Survey between 2003 and 2009, there were 4,098 (26%) adults who did not grant permission to obtain their hospital records or calculate their neighborhood greenness variables. A comparison between the age, sex, education, household income and BMI distributions for the study population and the excluded (non-linkable) population is provided in the Supplementary Material. Although the study population differed from the non-linkable population in terms of these variables, the extent of this difference was marginal.

## Discussion

A protective association was observed between levels and variability of neighborhood greenness and coronary heart disease or stroke. The odds of hospitalization was 37% lower, and the odds of self-reported heart disease or stroke was 16% lower, among adults with highly variable greenness around their home, compared to those in neighborhoods with low variability in greenness. These effects were independent of the absolute level of greenness in the neighborhood. We observed weaker evidence for association between the cardiovascular outcomes and mean neighborhood greenness. The odds of self-reported heart disease or stroke decreased by 7% per unit (interquartile range) increase in the level of greenness.

The lower prevalence of heart disease may be attributable to higher levels of physical activity, such as neighborhood walking which is positively influenced by the natural and built environment. Previous studies have reported that adults with access to a large high-quality park within walking distance (also 1600 m) from home have elevated levels of walking [9,17] and longevity [18]. Our results indicate that these results might be explained by neighborhood variability in greenness, which is more strongly associated with a lower risk of coronary heart disease or stroke. This suggests that in terms of cardiovascular health, the mix of greenness is more relevant than the extent of greenness. That is, both green and non-green areas are necessary within walking distance, rather than vegetation alone. Neighborhood attributes that may contribute to a high variability in greenness might include prevalence of tree lined streets/cycleways/footpaths, presence of parks with parking, or green reserves with good road connectivity. The coexistence of both aesthetically pleasing natural vegetation to entice people out of their homes and destinations within walking distance would also contribute to variability in neighborhood greenness. A review of the environmental influences on walking concluded that aesthetic neighborhood attributes (which included ratings of natural features) were found to be associated with walking [7]. Furthermore, a cross-sectional study conducted in Seattle, US, reported that the most frequently walked non-green destinations were grocery stores, restaurants, libraries, coffee shops, and post offices[19]. Further studies are needed to identify the specific attributes of neighborhoods with a high degree of variability in greenness, such as the co-location of green and non-green areas (e.g. tree-lined paths, parks surrounded by well-connected streets). Future cohort studies of coronary heart disease and stroke should include measurement of variability in neighborhood greenness.

A limitation of this study was that the mediating effects on physical activity were not directly examined because we did not have a measure of physical activity undertaken in the neighborhood. For example, neighborhood walking might be more appropriate to specifically test the hypothesis. The results of this cross-sectional study should be interpreted cautiously, as they might be explained by self-selection of healthier individuals into neighborhoods with high levels of variability in greenness.

Although this study was limited to a cross-sectional design, temporal relevance of hospitalizations was improved by ensuring that cases were defined if the hospitalization occurred within a 3 year window of the year of the health survey and calculation of neighborhood greenness. Adjustment for a wide range of risk factors, such as nutrition, was an advantage of this study. However, adjustment was not made for known risk factors for cardiovascular disease such as heritability, which were not available from the health survey. The possibility of residual confounding by individual level socio-economic status cannot be dismissed. However, adjustment was also made for multiple correlates of socioeconomic position, including education and household income. Adjustment was also made for biological and behavioral factors that also exhibit gradients in socioeconomic status.

A further limitation is that imagery obtained from remote sensing is accurate to a specified level of resolution. It is possible that the spatial variability in greenness could have been deflated for neighborhoods with smaller block sizes as an artifact of the spatial resolution of the imagery. However, smaller block sizes in our study area were typically located closer to the city with greater land-use mix. Therefore, we suspect that the deflation in spatial variability in greenness due to the effect of the resolution of the imagery was small relative to effect of actual variability in greenness and land-use mix. Finally, spatial autocorrelation in cardiovascular outcomes was not directly modeled in the analyses. However, such autocorrelation would be limited to that not already accounted by adjustment for socioeconomic factors (e.g. education and household income) which also cluster spatially and are strong predictors for cardiovascular disease. Perhaps more importantly, it is impossible to completely rule out the chance of spatial autocorrelation in the cardiovascular outcomes due to an unknown factor.

## Conclusions

Greater variability in greenness about the home was associated with lower odds of self-reported prior diagnoses and hospitalization with coronary heart disease or stroke. These effects were independent of the mean level of greenness and suggest a mediating influence of neighborhood variability in greenness on promoting physical activity. Future cohort studies should include a measure of variability in neighborhood greenness.

## Competing interests

The authors declare that they have no competing interests**.**

## Authors’ contributions

GP conceived the study, conducted the statistical analysis and drafted the manuscript. All authors were involved in redrafting of the manuscript, provided advice on re-analyses for successive drafts, and interpreted the results. All authors read and approved the final manuscript.

## Pre-publication history

The pre-publication history for this paper can be accessed here:

http://www.biomedcentral.com/1471-2458/12/466/prepub

## Supplementary Material

Additional file 1 **Supplementary Material [**[20]**].** Click here for file
