# Supplementary material for: The association between neighborhood greenness and cardiovascular disease: an observational study
Source: BMC Public Health. 2012 Jun 21;12:466. doi: 10.1186/1471-2458-12-466 (PMC3476430; doi:10.1186/1471-2458-12-466)
Supplement: Additional file 1 — Supplementary Material [[20]]. [file 1471-2458-12-466-S1.doc]

**Supplementary Material**

**Categorization of NDVI**

This document is a description of the exploratory process by which we arrived at the decision to categorize NDVI into tertiles. The process was as follows:

1. We separately plotted the event risk against SD NDVI and against mean NDVI. As the event was a binary outcome, we applied a loess smoother (i.e. a nonparametric smoothing function that performs localized regression for a given value of the smoothing parameter) and then transformed these values to the log(odds) scale.

2. We looked for non-linearity in these plots for the region between the 10th percentile (P10) and the 90th percentile (P90) i.e. for the bulk of the data.

3. We repeated the above steps for a range of values of the smoothing parameter.

4. Visual assessment of both the figure and the density enabled us to select cut-points to ensure sufficient data in each category.

Figures 4 and 5 show both the log(odds) of the loess smoothed risk as well as the density of the greenness variable for the standard deviation of NDVI and the mean NDVI respectively. Both figures indicate an irregular pattern between the P10 and P90. For both figures, we inferred that the pattern may be approximated parsimoniously by a piecewise constant model. Cut-points at tertiles (P33 and P67) seemed to provide a compromise between capturing the pattern of the association and ensuring sufficient data within each category. We did not use the data outside the P10-P90 range to inform our choice of cut-points because of the potential for edge-effects i.e. patterns that are an artifact of insufficient data at the lowest and highest extents of the greenness variable.

Tertiles also provide an equal amount of data in each category and allow interpretation relative to “low”, “medium” and “high” values. The effects of extreme (small and large) greenness values (i.e. outliers) can also be reduced by such categorization. Also, odds ratio estimates cannot be as readily extracted had we opted for higher order polynomial terms instead.

**Figure 4:** Log odds of the loess smoothed outcome (self-reported heart disease or stroke), kernel density, and standard deviation of NDVI. The 10^th^ (P10) and 90^th^ (P90) percentiles are illustrated along with the low (P33) and high (P67) tertiles. The figure illustrates potential for two distinct levels of the log odds within the middle 80% of the data.


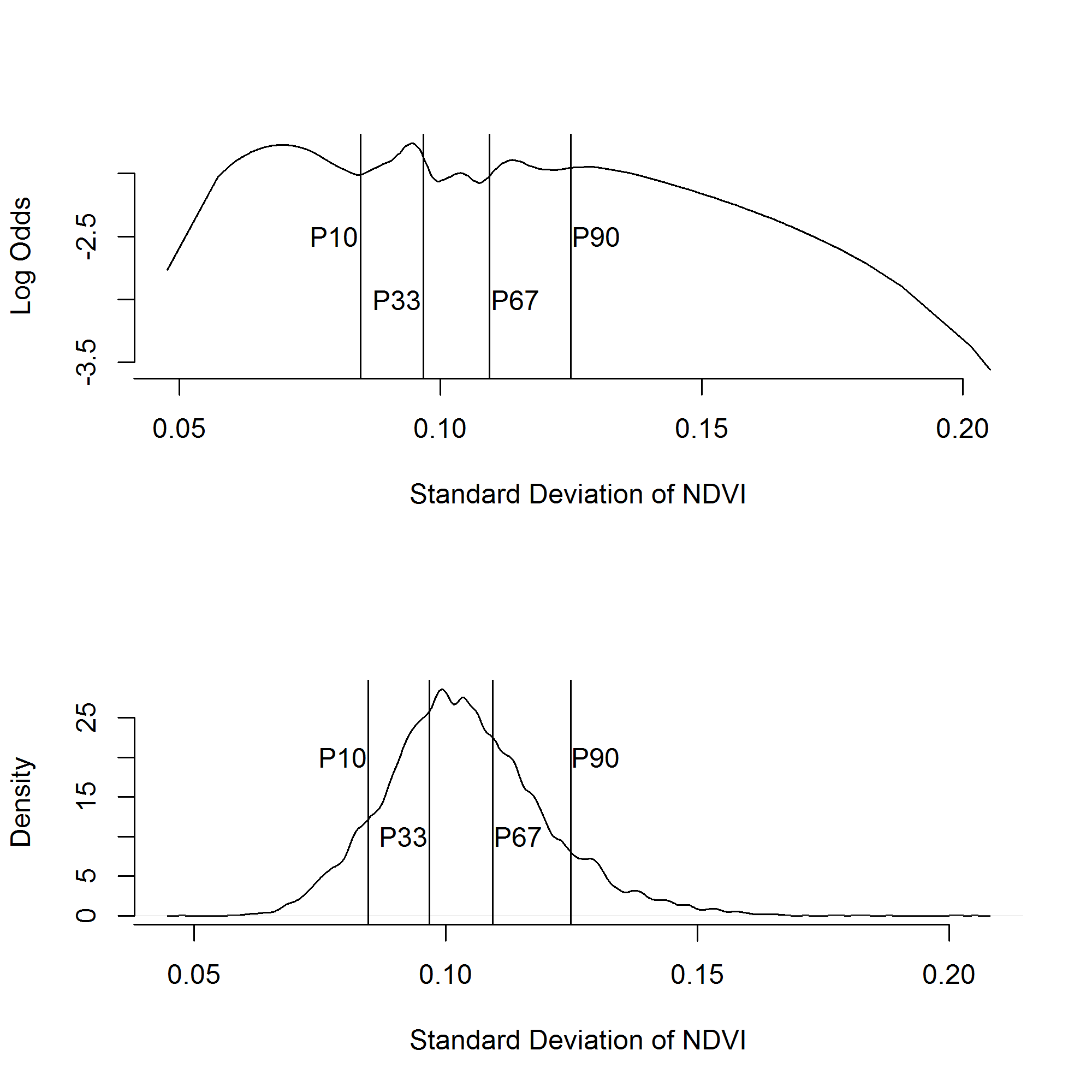


**Figure 5:** Log odds of the loess smoothed outcome (self-reported heart disease or stroke), kernel density, and mean NDVI. The 10^th^ (P10) and 90^th^ (P90) percentiles are illustrated along with the low (P33) and high (P67) tertiles. The figure illustrates potential for three distinct levels of the log odds within the middle 80% of the data.


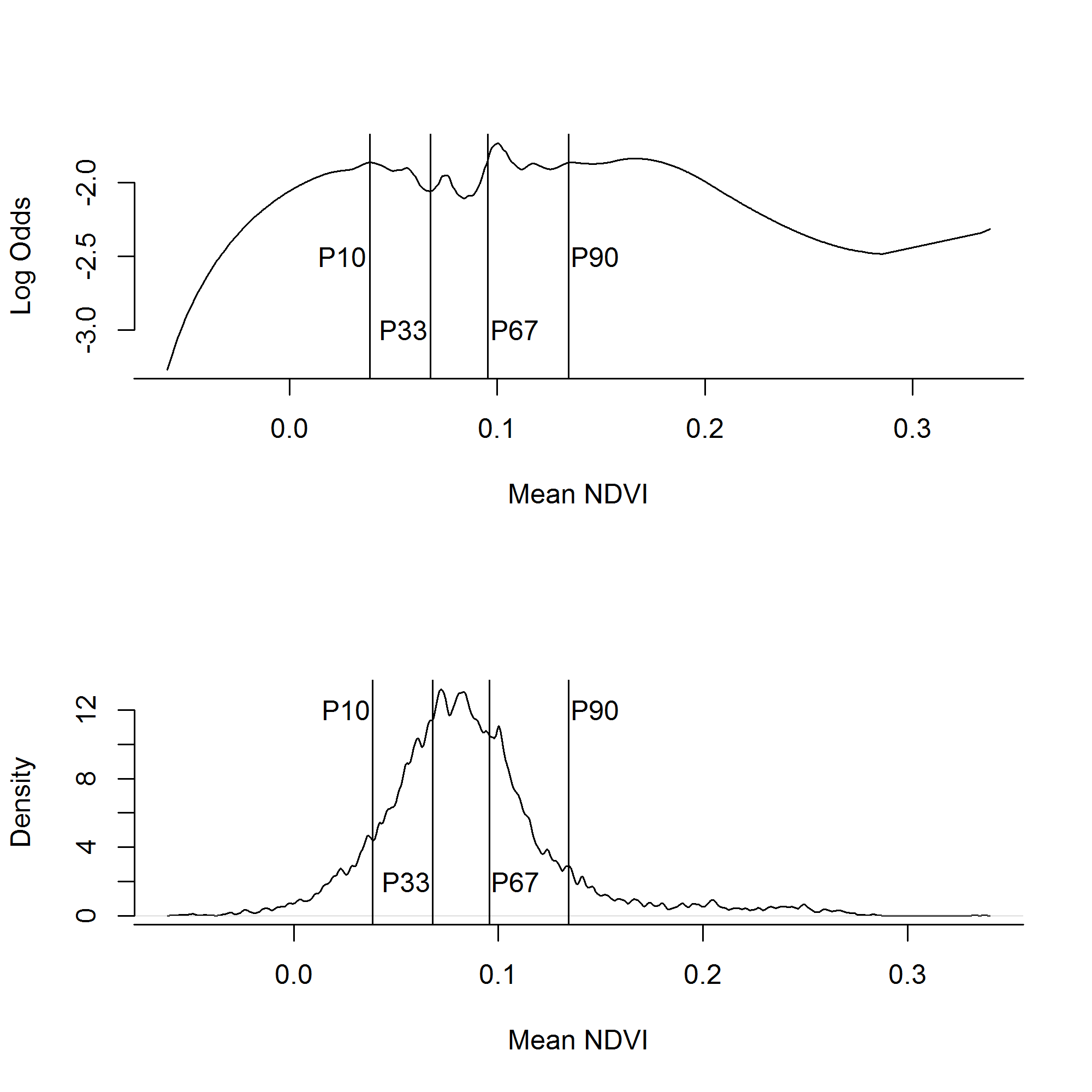


**Assessment of external generalizability**

There were 15,502 adult residents in Perth who completed the survey between 2003 and 2009. Of those participants, 11,404 (74%) participants granted permission for data linkage; the “study population”. That is, responses to the Health and Wellbeing Survey were available for the 26% (N=4098) of adults who completed this survey but did not grant permission for us to obtain their hospital records or calculate their neighbourhood greenness variables; the “nonlinkable population”.

The nonlinkable population had a similar age and sex distributions to those included in the study except that they were slightly younger (Figure 6) and more likely to be female (Figure 7). The adults in the nonlinkable population were more likely to have completed the last year of secondary school as their highest level of education (Year 12) and were less likely to have a trade qualification (Figure 8). The household income distributions for the study population and the nonlinkable population were indistinguishable (Figure 9). The nonlinkable population had slighter lower BMIs than the study population (Figure 10). In general, the adults in the study population were more likely to possess risk factors for cardiovascular disease than those who did not agree to data linkage. Consequently, they were more likely to have reported coronary heart disease or stroke (Figure 11). However, the Chisquare and Kolmogorov-Smirnov tests are highly sensitive to differences between distributions. The figures indicate that the shape of the distributions were similar. Moreover, the difference between the centers of the distributions (for continuous outcomes) and frequencies (for categorical outcomes) were small, albeit “statistically” significant.

**Figure 6:** Age distribution of adults in the study population and those not included in the study (nonlinkable population), with Kolmogorov-Smirnov test for equality of distributions.


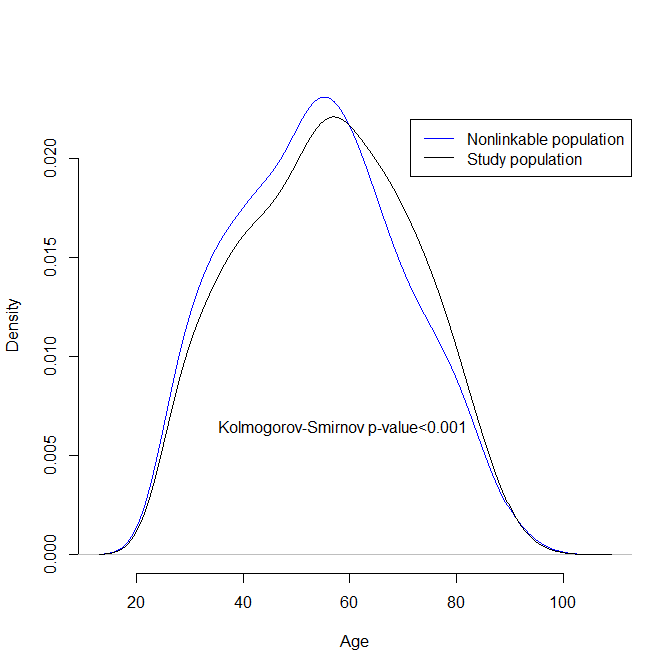


**Figure 7:** Sex distribution of adults in the study population and those not included in the study (nonlinkable population), with Chisquare test for equality of distributions.


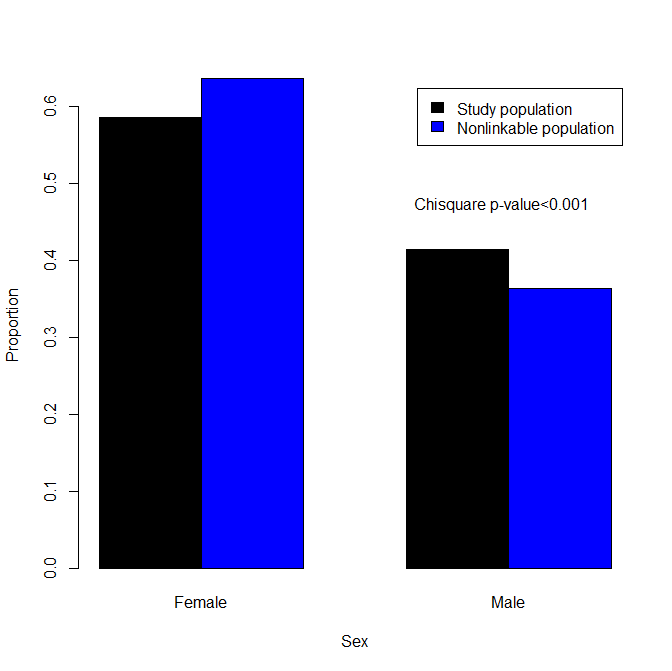


**Figure 8:** Education distribution of adults in the study population and those not included in the study (nonlinkable population), with Chisquare test for equality of distributions.


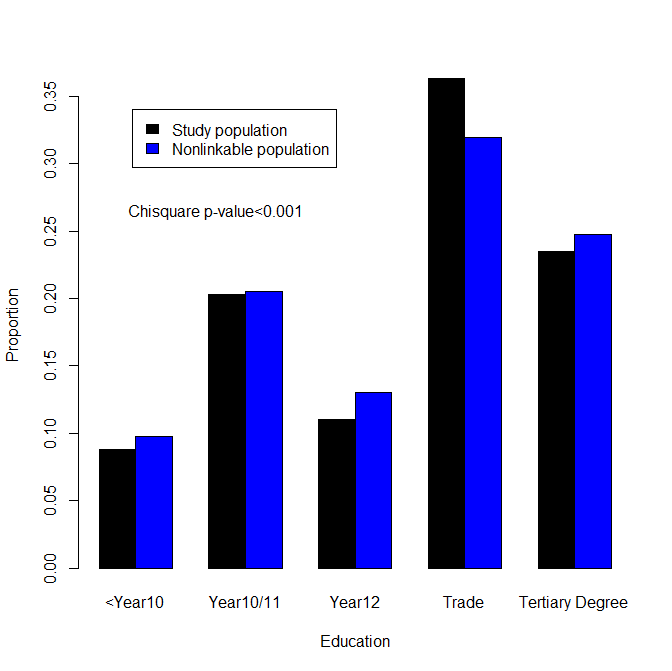


**Figure 9:** Household income distribution of adults in the study population and those not included in the study (nonlinkable population), with Kolmogorov-Smirnov test for equality of distributions.


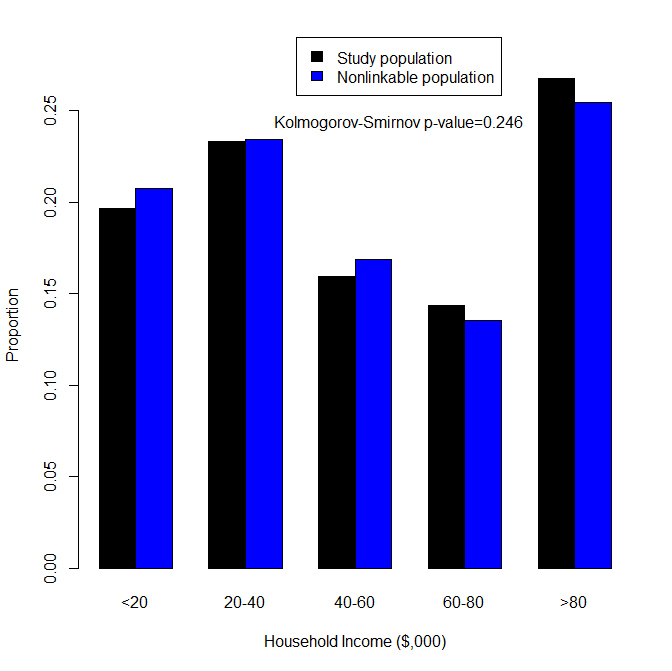


**Figure 10:** BMI distribution of adults in the study population and those not included in the study (nonlinkable population), with Kolmogorov-Smirnov test for equality of distributions.


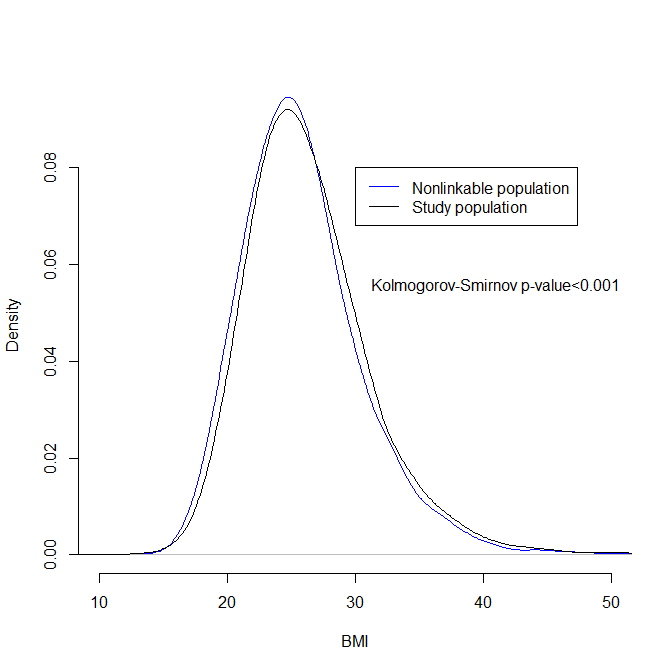


**Figure 11:** Distribution of self-reported heart disease or stroke for adults in the study population and those not included in the study (nonlinkable population), with Chisquare test for equality of distributions.

**
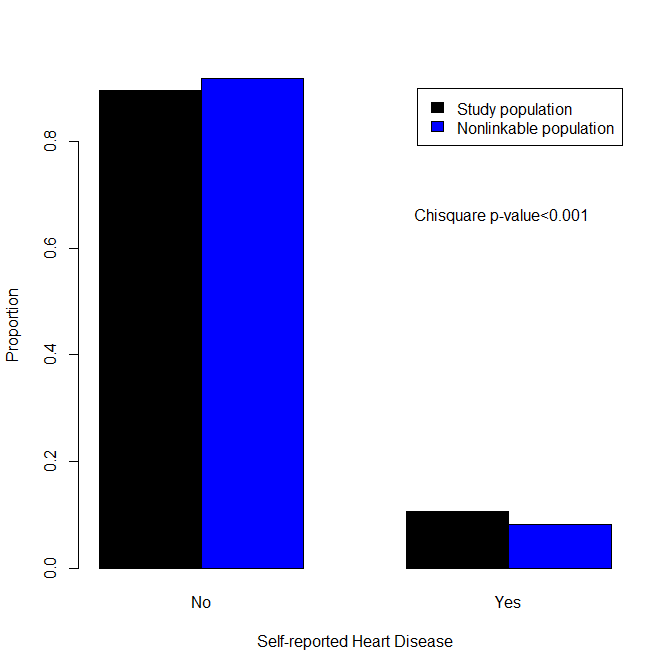
**

**References**

1. W. S. Cleveland, E. Grosse and W. M. Shyu (1992) Local regression models. Chapter 8 of Statistical Models in S eds J.M. Chambers and T.J. Hastie, Wadsworth & Brooks/Cole.
